# Conceptualization of the Holobiont Paradigm as It Pertains to Corals

**DOI:** 10.3389/fphys.2020.566968

**Published:** 2020-09-23

**Authors:** Tamar L. Goulet, Ivan Erill, Marina S. Ascunce, Sheree J. Finley, Gulnaz T. Javan

**Affiliations:** ^1^Department of Biology, University of Mississippi, University, MS, United States; ^2^Department of Biological Sciences, University of Maryland Baltimore County, Baltimore, MD, United States; ^3^Emerging Pathogens Institute, University of Florida, Gainesville, FL, United States; ^4^Plant Pathology Department, University of Florida, Gainesville, FL, United States; ^5^Department of Physical Sciences and Forensic Science Programs, Alabama State University, Montgomery, AL, United States

**Keywords:** coral reefs, symbiosis, mutualism, Symbiodiniaceae, microbiome, holobiont

## Abstract

Corals’ obligate association with unicellular dinoflagellates, family Symbiodiniaceae form the foundation of coral reefs. For nearly a century, researchers have delved into understanding the coral-algal mutualism from multiple levels of resolution and perspectives, and the questions and scope have evolved with each iteration of new techniques. Advances in genetic technologies not only aided in distinguishing between the multitude of Symbiodiniaceae but also illuminated the existence and diversity of other organisms constituting the coral microbiome. The coral therefore is a meta-organism, often referred to as the coral holobiont. In this review, we address the importance of including a holistic perspective to understanding the coral holobiont. We also discuss the ramifications of how different genotypic combinations of the coral consortium affect the holobiont entity. We highlight the paucity of data on most of the coral microbiome. Using Symbiodiniaceae data, we present evidence that the holobiont properties are not necessarily the sum of its parts. We then discuss the consequences of the holobiont attributes to the fitness of the holobiont and the myriad of organisms that contribute to it. Considering the complexity of host-symbiont genotypic combinations will aid in our understanding of coral resilience, robustness, acclimation, and/or adaptation in the face of environmental change and increasing perturbations.

## Introduction

Linnaeus hypothesized that gorgonian corals were plants which metamorphosed into animals. In 1775, John Ellis wrote to Daniel Solander (Ellis, 1776) who requested “that I (Ellis) should continue my researches into the formation and growth of … Gorgonia… known in English by the name of sea fans, sea feathers, and sea-whips… This you thought the more necessary, as the accounts already published of them by the illustrious Dr. Linnaeus and Dr. Pallas seemed to make them of a mixed nature in their growth, between animals and vegetables…” After studying gorgonian morphology, Ellis (1776) concluded: “…that though they grow in a branched form, they are no more allied to vegetables… that animal life doth not depend on bodies growing according to a certain external form.”

Although Ellis debunked the hypothesis that a coral changed from a plant to an animal, a coral (either a scleractinian coral or an octocoral, herein referred to collectively as coral) is actually a coral consortium, which includes not only the coral animal but also dinoflagellates, apicomplexans, fungi, bacteria, and *Archaea* (reviewed in Knowlton and Rohwer, 2003; Olson and Kellogg, 2010; Blackall et al., 2015; Leggat et al., 2019). We posit that to understand corals, and by extension coral reefs, it is imperative to acknowledge and incorporate the role of the consortium in shaping the coalesced characteristics. Such a holistic approach assesses the combined coral entity, the coral holobiont.

Lynn Margulis defined a biont as an “individual organism,” and a holobiont as a “symbiont compound of recognizable bionts” (Margulis, 1991). Although members of a holobiont interact in a symbiosis, not every symbiotic organism is part of a holobiont. In this review, we define the coral holobiont as containing the coral and the microbiota found within the coral body, its mucus, and its skeleton, a definition in line with that of Rohwer et al. (2002). We exclude organisms that reside in the immediate vicinity of corals, such as crabs and shrimp (Glynn, 1980) or coral dwelling fish (Liberman et al., 1995), even if they engage in mutualisms with corals.

## The Coral Holobiont Constituents

At the core of the coral reef ecosystem is the obligatory symbiosis between corals and dinoflagellate algae, family Symbiodiniaceae. This mutualism relies on “access to metabolic capabilities” and “protection from antagonists” (Douglas, 2010). The coral gains photosynthetically fixed products from the Symbiodiniaceae (Muscatine and Porter, 1977). In scleractinian corals, Symbiodiniaceae also enhance coral calcification (Goreau and Goreau, 1959; Pearse and Muscatine, 1971). Symbiodiniaceae uptake the coral’s nitrogenous wastes (Muscatine and D’Elia, 1978), a valuable commodity in the oligotrophic seas, where coral reefs occur. As endosymbionts, the Symbiodiniaceae gain a degree of protection from both environmental conditions and predators (Douglas, 2010).

Knowledge about the rest of the coral holobiont microbiota lags behind information about Symbiodiniaceae. The second most studied component is the bacterial consortium. The coral enables “access to metabolic capabilities” *via* the wax ester and triglycerides in its mucus (Johannes, 1967; Benson and Muscatine, 1974). Bacteria may provide carbon, nitrogen, and sulfur (reviewed in Shashar et al., 1994; Knowlton and Rohwer, 2003; McDevitt-Irwin et al., 2017) and “protection from antagonists” by producing antibiotics and cell-to-cell communication inhibitors, inhibiting swarming, and through their own growth, outcompeting and preventing other microbes from settling on the coral (reviewed in McDevitt-Irwin et al., 2017; Peixoto et al., 2017). Data on the remainder of the holobiont consortium are sparse. The *Archaea* may be involved with nitrogen cycling, the viruses potentially with gene transfer, and the fungi may protect from environmental conditions, provide antimicrobial activity, and take part in carbon and nitrogen cycles (reviewed in Knowlton and Rohwer, 2003; Peixoto et al., 2017).

## The Identity of the Holobiont Partners is Progressively Revealed in Cyclical Waves

A fundamental aspect to understanding holobionts is partner identification. For many consortium members, their small sizes, lack of morphological differences, and morphological plasticity, severely limited their identification (Wilcox, 1998). Advancements in genetic techniques, alongside cost reduction, have progressively enabled identifying the holobiont partners. Knowledge gains have occurred incrementally, as finer levels of resolution become possible, and gains grow in cyclical waves, whereby identification of one of the partner groups commands center stage, followed by the next. In coral holobionts, Symbiodiniaceae identification led the way.

Brandy in 1881 referred to the dinoflagellates as zooxanthellae (cited in Blank and Trench, 1986). Zooxanthellae were once attributed to one pandemic species, *Symbiodinium microadriaticum* (Freudenthal, 1962). Distinguishing between zooxanthellae took off in the early 1990s when utilizing differences in the nuclear genes that encode small ribosomal subunit RNA, led to placement of zooxanthellae into several groups (Rowan and Powers, 1991a, 1992), later referred to as *Symbiodinium* clades (Baker and Rowan, 1997; Baker et al., 1997; Goulet and Coffroth, 1997), a term used for the next 21 years. Other DNA regions provided within-clade, population, and individual level resolution (reviewed in Goulet et al., 2019). Only recently were these dinoflagellates placed in the family Symbiodiniaceae (LaJeunesse et al., 2018).

The feasibility of genetically distinguishing between Symbiodiniaceae led to a characterization frenzy. Numerous publications presented and/or synthesized the data available on Symbiodiniaceae genera and species identities in coral species on mesophotic reefs around the world (Goulet et al., 2019), and in different geographic locations (LaJeunesse, 2002; Savage et al., 2002; LaJeunesse et al., 2003, 2004a,b, 2008; Goulet and Coffroth, 2004; van Oppen et al., 2005; Putnam et al., 2012; Ziegler et al., 2019). With the reduction in sequencing costs, techniques involving visualization of Symbiodiniaceae DNA fragments *via* gels (Rowan and Powers, 1991a,b; Belda-Baillie et al., 1999; LaJeunesse, 2001) have given way to direct sequencing (e.g., Arif et al., 2014). The burst of Symbiodiniaceae genetic characterization is now mirrored in the bacterial component of the coral holobiont. Starting a decade later, the prevailing approaches utilize the 16S ribosomal RNA (rRNA) as a canonical biomarker. Although on a smaller scale, bacterial consortia in corals have been identified from different geographic locations (reviewed in Blackall et al., 2015; McDevitt-Irwin et al., 2017; van de Water et al., 2018) and mesophotic reefs (Olson and Kellogg, 2010; Leggat et al., 2019). The application of high-throughput amplicon and metagenomic analyses enables not only the characterization of bacteria but also of the other microbial partners such as fungi, *Archaea*, and viruses (reviewed in Wegley et al., 2007; Blackall et al., 2015; Góes-Neto et al., 2020).

## The Specificity of the Coral Holobiont and the Ramifications of Transient Entities

**Figure 1:**
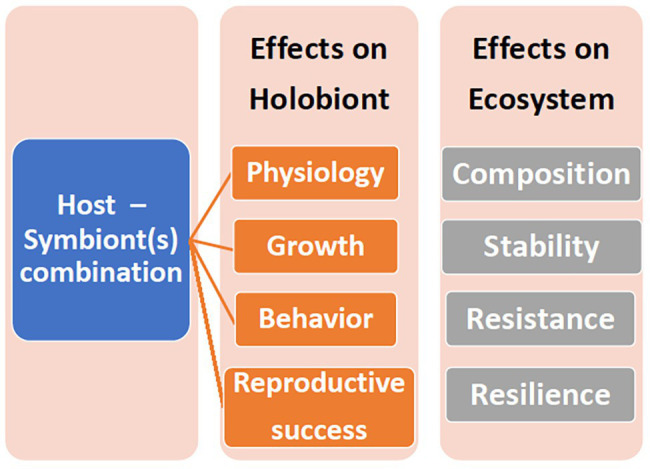
The ramifications of different host-symbiont genotypic combinations on the holobiont and the ecosystem.

A holobiont is a conglomerate of entities, representing multiple phyla. If components of this consortium leave, or new entities enter, then even the same coral colony may represent a different holobiont at different times and/or under different environmental conditions. Coral species host specific Symbiodiniaceae genera and types, and these do not change even under stressful conditions (Goulet, 2006, 2007), although shuffling of the proportion of existing types may occur (Berkelmans and van Oppen, 2006), as well as transient or low level Symbiodiniaceae (Silverstein et al., 2012). Likewise, bacterial specificity exists in corals (Ainsworth et al., 2015; Shirur et al., 2016; van de Water et al., 2017, 2018; Huggett and Apprill, 2019; McCauley et al., 2020). Bacteria may consistently inhabit the holobiont (“core microbiome”) or be transient (Hernandez-Agreda et al., 2017; van de Water et al., 2017; Leite et al., 2018) and/or vary geographically (Osman et al., 2020). Furthermore, the coral itself may host different bacteria in the surface mucus layer (SML), tissue, or skeleton (Ainsworth et al., 2010; Gajigan et al., 2017). The role of transient or low abundant entities is speculated. The Coral Probiotic Hypothesis, for example, relies on the premise that transient or low level bacteria become pronounced (Reshef et al., 2006). The concept of specificity commands attention since, whether the holobiont entity can change or not affects interpretation of coral acclimation, adaptation, resilience, and persistence (Figure 1).

## Discussion

### A Holistic Approach Requires Evaluating the Entire Holobiont, Abandoning Interpretation Based on Only One of the Partners

Scientists approach the world through a personal prism formulated through research interests and academic experiences. Coral research may straddle multiple departments, either across taxonomic lines such as between departments of Zoology and Plant Sciences, or across resolution levels such as between Ecology vs. Cell and Molecular Biology departments. Departmental and training allegiances are nothing new. In 1931, Gardiner said “the investigator of the ‘coral reef problem’ to-day is usually either a geologist or a zoologist, for the botanist has not understood, up to the present, that he may claim an equal partnership” (Gardiner, 1931).

Terminology may also lead to biases in approaching holobiont related research questions. Since coral holobionts are often addressed in symbiotic terms, the coral is the host and the consortium constituents are referred to as the symbionts. The terms allude to size, the coral is the largest in the holobiont consortium; and to physical location, with the symbionts residing within or on the host. The words host and symbiont, however, are often extrapolated to include who controls whom. The dichotomy of interpretation was already articulated by Gardiner: “These special difficulties in respect to the nutrition of coral-building sedentary animals in tropical waters have been met by the ‘taming’ of unicellular green flagellates (Zooxanthellae) by the polyps – or the polyps being adopted by such plants-housing them as symbionts in their endoderm cells” (Gardiner, 1931).

Although data provide facts, an investigator’s approach affects the interpretation of those facts. Often it is assumed that the host controls the symbionts. Even though endosymbionts reside within a host, they can affect the host. For instance, the scleractinian corals *Acropora millepora* and *A. tenuis* can host/be inhabited by two Symbiodiniaceae genera, *Cladocopium*, and *Durusdinium*. Juvenile corals with *Cladocopium* grew up to three times faster than juveniles that hosted *Durusdinium* (Little et al., 2004). In a benthic organism, growing faster may increase survivorship, and coral size affects the onset of reproduction (Rinkevich and Loya, 1979). Therefore, the symbiont’s effect on coral growth affects holobiont survival and fitness.

Another example of how an approach affects interpretation pertains to corals and environmental perturbations. When corals encounter stressors, such as elevated seawater temperatures, a reduction of Symbiodiniaceae and/or their photosynthetic pigments can occur, a state termed “bleaching” (Glynn, 1996). Due to the obligate symbiosis between Symbiodiniaceae and many corals, bleaching may lead to holobiont death. It is still debated if algal loss is driven by the coral host expelling either dead algae or algae that impose a high metabolic demand (Weis, 2008), or if the algae instigate abandoning a sinking ship (Baird et al., 2009). Bleaching mechanisms may vary, and be holobiont-dependent and context-dependent. For example, in holobionts which host different Symbiodiniaceae genera and species, variation in conspecific bleaching may be attributed to the Symbiodiniaceae inhabiting the holobionts (Rowan, 2004; Goulet et al., 2005; Berkelmans and van Oppen, 2006; Sampayo et al., 2008). Conversely, in other coral species, no correlation exists between bleaching and the Symbiodiniaceae identity (Goulet et al., 2008), and the coral’s response to the perturbation may affect the bleaching outcome for the holobiont (Baird et al., 2009).

Although coral bleaching is considered a consequence of stress, the Adaptive Bleaching Hypothesis (ABH) contemplated that bleaching may be beneficial (Buddemeier and Fautin, 1993). *Via* bleaching, Symbiodiniaceae not optimal for new environmental conditions could be lost. Novel Symbiodiniaceae, better suited for the new conditions, could enter the holobiont from the environment. The ABH article recognized that holobiont physiology arises from “characteristics of the combination, rather than of the host or symbiotic algal partner alone” (Buddemeier and Fautin, 1993). Subsequent statements, however, such as “Bleaching provides an opportunity for the host to be repopulated with a different type of partner…” (Buddemeier and Fautin, 1993) turned this hypothesis into a coral-centric scenario. ABH being coral driven has been echoed in the literature (e.g., Baker et al., 2015). Although portraying phenomena with neutral, non-directional words (e.g., loss vs. expulsion of algae) is difficult, one needs to be cognizant of how word choice affects interpretation.

### A Holistic Approach Requires the Realization That Holobionts Cannot Be Characterized Based Solely on One Component of the Holobiont

To achieve order, we label biological entities and try to assign them to categories. For example, scientists have termed certain coral species as hardier in their ability to withstand environmental perturbations than others (Loya et al., 2001). Likewise, blanket statements have been made about Symbiodiniaceae genera, such as that *Durusdinium* (previous clade D) is a thermally tolerant genus (Stat et al., 2013). *Durusdinium trenchii* is indeed heat tolerant (LaJeunesse et al., 2014; LaJeunesse, 2017). Overarching generalizations, however, such as if corals that host *Durusdinium* or acquire *Durusdinium* will preferentially survive climate change (Baker et al., 2004; Oliver and Palumbi, 2009; Stat and Gates, 2011; Stat et al., 2013), may lead to misconceptions. Not all *Durusdinium* are heat tolerant and some inhabit only certain specific coral hosts (LaJeunesse et al., 2014). Therefore, attributing characteristics such as “thermal tolerant” should be done at the holobiont level rather than focusing on partner attributes.

### A Holistic Approach Requires Investigating Holobiont Characteristics and Holobiont Performance

The holobiont is a product of the interactions between the partners. Focusing on one partner, especially in isolation, is informative, although limited for understanding the holobiont. Cultured Symbiodiniaceae can provide information on the physiological range and attributes of Symbiodiniaceae and enable between‐ and within‐ Symbiodiniaceae comparisons (Banaszak et al., 2000; Tchernov et al., 2004; Robison and Warner, 2006; Brading et al., 2013). Culturable Symbiodiniaceae, however, may not represent the common Symbiodiniaceae found within the holobiont or may be a contaminant that does not occur in the holobiont (Goulet and Coffroth, 1997; Santos et al., 2001). Furthermore, *in hospite*, with the other partners present, the Symbiodiniaceae may exhibit a different physiology than what is observed in culture (Goulet et al., 2005). The host-symbiont genotypic combinations may also affect the physiology and ecology of the holobiont (Figure 1). The panmictic sea anemone *Exaiptasia pallida* (previously *Aiptasia pallida*) predominantly occurs with the Symbiodiniaceae genus *Breviolum*, although in Florida, it also hosts *Symbiodinium sensu stricto* and occasionally, *Cladocopium* (Thornhill et al., 2013). When exposed to elevated sea water temperatures, the Florida anemone – *Symbiodinium* holobionts had higher oxygen fluxes compared to Bermuda anemones with their natal *Breviolum* algae (Goulet et al., 2005). A lab produced host-symbiont genotypic combination of Bermuda anemones with *Symbiodinium* algae yielded a higher oxygen flux than either of the natal host-symbiont combinations at both 32 and 34°C (Goulet et al., 2005).

### A Holistic Approach Requires Assessing Parameters of as Many of the Holobiont Participants as Possible

Studying corals from a holobiont perspective benefits from measuring parameters of multiple consortium members. Case in point, the definition of coral bleaching does not distinguish what drives the Symbiodiniaceae loss. Two potential, not mutually exclusive, scenarios may occur. Coral cells can lose the Symbiodiniaceae cells within them, or, the stressor may lead to loss of coral cells, and since the host cells contain Symbiodiniaceae, the end result is that the coral has less Symbiodiniaceae within it. The common approach of assessing Symbiodiniaceae density (Fitt et al., 2000; Siebeck et al., 2006; Johnson and Goulet, 2007) does not address the two scenarios. Conversely, quantifying both algal and host parameters can illuminate the route to the bleaching outcome. For example, branches of the octocorals *Eunicea tourneforti* and *Pseudoplexaura crucis* exposed in the summer to sea water temperatures 3°C above ambient, lost Symbiodiniaceae (McCauley et al., 2018). In *E. tourneforti*, Symbiodiniaceae density fell 26%, and the number of Symbiodiniaceae normalized to holobiont lipid content (Shirur et al., 2014) also differed between ambient and elevated temperatures (McCauley et al., 2018). In *P. crucis*, the 35% reduction in Symbiodiniaceae density occurred alongside a 19% reduction in the lipid amount, resulting in no significant changes to Symbiodiniaceae numbers normalized to holobiont lipid content (McCauley et al., 2018). Thus, in *E. tourneforti*, lower Symbiodiniaceae density was driven by less Symbiodiniaceae per host cells, while in *P. crucis*, the drop in Symbiodiniaceae was accompanied by a reduction in host cells. Without collecting data on both host and symbiont parameters, the different routes that led to the same outcome would have been missed.

### A Holistic Approach Will Strive to Assess Whether Changes in the Holobiont Consortium Occurred During the Course of a Study

When attributes of conspecific holobionts differ temporally, spatially or due to perturbations, these differences may demonstrate acclimation and/or adaptation of the existing holobiont consortium. Alternatively, acclimation, and/or adaptation may manifest themselves in changes in a constituent of the genotypic complement of the holobiont. For example, in seven Caribbean octocoral species, sampled either seasonally or when exposed to perturbations, the Symbiodiniaceae did not change, nor did the dominant bacterial operational taxonomic units, although shifts in bacterial abundances occurred (McCauley et al., 2020). Similarly, in thermal stress experiments on *Acropora digitifera* in the Philippines (Gajigan et al., 2017) and *Acropora muricata* in Taiwan (Lee et al., 2015), the overall microbial community remained stable, with shifts in bacterial abundance in the tissue and SML. In six Red Sea coral species, Symbiodiniaceae specificity occurred throughout latitudinal sampling, while the bacterial composition and diversity in the SML varied (Osman et al., 2020), illustrating the importance of identifying the holobiont consortium and its specificity.

## Future Research Directions

### The Role of the Partners in the Holobiont

Even though identifying the holobiont partners is important, identification is primarily descriptive and correlative. The next step, where feasible, is to decipher the role consortia members play in the holobiont, from investigating holobiont ecology and physiology, assessing metabolic products produced and exchanged, to gene expression. Furthermore, roles may vary depending on environment and interactions with other holobiont members (Figure 1). Although many studies manipulated environmental parameters, such as temperature, and evaluated the outcome for corals, often the Symbiodiniaceae are not identified (McLachlan et al., 2020). Studies on parameter effects on coral holobiont – bacterial and *archaea* interactions lag further behind. As the field moves beyond identifying the microbiota complement of the holobiont, more experimental studies will ensue.

### Mechanisms Underlying Specificity and/or Flexibility of the Holobiont Consortium

In corals, a Symbiodiniaceae ontogenetic acquisition window appears to exist, after which Symbiodiniaceae specificity sets in (Coffroth et al., 2001; Weis et al., 2001). The mechanism that drives Symbiodiniaceae specificity and why coral species contain specific bacteria over other bacteria is unknown. The quandary of specificity vs. flexibility raises the issue of the definition of a holobiont from a different perspective, and that is whether entities that are transient in the coral consortium should be regarded as part of the holobiont? To address this point, what is considered transient needs to be defined.

### Is the Coral Holobiont the Unit of Selection?

A coral holobiont exhibits characteristic physiological and ecological traits. These characteristics not only separate one coral species from another, but also conspecifics coral-symbiont genotypic combinations. The physiological performance of Symbiodiniaceae, for example, differs in different hosts. If the traits of a coral holobiont are driven by the holobiont consortium, is the holobiont the unit of selection? Does the holobiont depend on its specific consortia, or just on their physiological roles? And, does theoretical modeling, for example, coral survival under different climate conditions, require incorporating holobiont variables?

## Conclusion

The coral holobiont is a consortium of phylogenetically disparate entities co-existing in a coral. The holobiont concept adds a level of complexity in deciphering the ecology and evolution of corals. To address research questions pertaining to coral holobionts, researchers need to leave research silos defined by research organisms, research training, and departmental affiliation. Although characterizing multiple aspects of a coral holobiont may not be feasible, considering that the data may arise from the myriad of participants in the holobiont may affect data interpretation. Due to the diversity of the coral holobiont consortium, collaborations of multiple investigators with multiple skill sets and knowledge about the multiple components of the coral holobiont will be key.

## Author Contributions

TLG, IE, MSA, SJF, and GTJ contributed to the conception of the review. TLG wrote the first draft of the manuscript. TLG, IE, MSA, SJF, and GTJ contributed to manuscript revision, read and approved the submitted version.

### Conflict of Interest

The authors declare that the research was conducted in the absence of any commercial or financial relationships that could be construed as a potential conflict of interest.
